# Characterization and optimization of extracellular enzymes production by *Aspergillus niger* strains isolated from date by-products

**DOI:** 10.1186/s43141-021-00145-y

**Published:** 2021-03-31

**Authors:** Reda Bellaouchi, Houssam Abouloifa, Yahya Rokni, Amina Hasnaoui, Nabil Ghabbour, Abdelkader Hakkou, Abdelmajid Bechchari, Abdeslam Asehraou

**Affiliations:** 1 Laboratory of Bioresources, Biotechnology, Ethnopharmacology and Health, Faculty of Sciences, Mohammed Premier University, 60 000 Oujda, Morocco; 2 National Institute of Agronomic Research (INRA), Oujda Center, 60 000 Oujda, Morocco

**Keywords:** Extracellular, Enzymes, Date by-products, *Aspergillus niger*

## Abstract

**Background:**

This work aims to study the optimal conditions of the fermentation culture medium used for the production of extracellular enzymes (amylase, cellulase, lipase, and protease) from previously isolated *Aspergillus niger* strains in date by-products.

**Results:**

The five most powerful isolates selected based on the zone of degradation formed on Petri plates by the substrate were subjected to the quantitative evaluation of their enzymatic production. All five strains showed almost similar API-ZYM profiles, with minor variations observed at the level of some specific enzyme expression. The production of cellulase and amylase was depending on pH and incubation temperatures. ASP2 strain demonstrated the high production rate of amylase (at pH 5 and 30 °C) and cellulase (at pH 6 and 30 °C) for 96 h of incubation.

**Conclusion:**

The *A. niger* showed the ability to produce several extracellular enzymes and can be used in the valorization of different agroindustrial residues.

## Background

*Aspergillus niger* is the most commonly used industrial *Aspergillus* species for the production of pharmaceuticals, food ingredients, and enzymes [1, 2]. It was the most important fungi used worldwide for biotechnological applications [3]. *A. niger* is known for its capacity to produce a broad range of enzymes related to the degradation of plant polysaccharides, such as cellulose, xylan, xyloglucan, galactomannan, and pectin [4]. These enzymes are essential to convert the natural carbon sources of these fungi (mainly plant polymers) into small molecules that can be taken up into the cell and can be widely used in the industry [4].

Considering the substantial availability of highly rich date by-products at very low prices, the use of these wastes as raw material for different bioproduction could provide a profitable substrate for low-cost biotechnological productions. These date fruits are mainly composed of sugars, a low percentage of fat and protein [5, 6], and significant amounts of total dietary fibers (TDF) (~ 7.81–13.63% dry matter basis) [7]. *A. niger* strains isolated from this environment should have the necessary enzymatic activity to degrade these elements to produce their requirements in terms of carbon, nitrogen, vitamins, and amino acids.

As we know, the production and yield of the enzyme were influenced by many factors, including temperature, pH, carbon, and nitrogen source [8, 9]. The purpose of this study is the characterization of enzymatic production in different environmental conditions (incubation time, pH, and T °C), using *Aspergillus niger* strains, isolated from undervalued dates and processed date by-products [5].

## Methods

### Fungal strains and culture conditions

A total of 40 strains of *Aspergillus niger* used in this work were isolated from date by-products and identified in previous work [5]. One millimeter of each diluent was plated on the solidified PDA plates (Biokar, France) by streaking. The plates were incubated at 25 °C for 3–5 days to ensure maximum fungal growth. Characteristic growth of *A. niger* (initially white, rapidly turning black) was subjected to microscopic observation. For microscopic identification, a drop of lactophenol blue was placed on a clean slide, with a fragment of the fungal growth, and observed under the microscope using a ×10 then ×40 objectives. The isolates obtained were given a number preceded by ASP. In this way, 40 isolates were purified and stored on inclined tubes of the PDA medium at 4 °C until they were used. These strains were routinely reactivated and cultured in PDA at 25 °C for 7 days before use.

### Screening extracellular enzymes from *A. niger*

#### Cellulase activity

The cellulase activity of *A. niger* strains was realized in Czapek-Dox agar medium containing sucrose 30 g/L, sodium nitrate 3 g/L, magnesium sulfate 0.5 g/L, potassium chloride 0.5 g/L, iron(III) sulfate 0.01 g/L, dipotassium hydrogen phosphate 1 g/L, agar 12 g/L, and carboxymethylcellulose (CMC) 1% (w/v) (Sigma-Aldrich, USA). *A. niger* strains were inoculated with 5 mm of the mycelium at the center of the plate and incubated for 5 days at 25 °C. After incubation, the cultures were flooded with a Congo red solution (0.2%) and then bleached with 1M NaCl for 15 min. Clear zones obtained around the fungal colony indicated cellulolytic activity. All experiments were realized in triplicate.

#### Amylase activity

The amylase activity was evaluated by measuring the ability of *A. niger* strains to hydrolyze starch in the agar medium. 5 mm of mycelia from strains was placed in the YPD medium containing dextrose 1g/L, yeast extract 0.1g/L, peptone 0.5 g/L, and agar 16 g/L, and supplemented with 1% (w/v) of soluble starch (Sigma-Aldrich, USA). After incubation at 25 °C for 5 days, the plates were flooded with a solution containing 1% iodine solution in 2% potassium iodide. Zone of clearance around the colony indicated amylase activity and was measured. The test was performed in triplicate.

#### Lipase activity

The lipase activity was detected in a medium containing peptone 10 g/L, NaCl 5 g/L, CaCl_2_·2H_2_O 0.1 g/L, and agar 16 g/L and autoclaved at 121 °C for 20 min. Ten milliliters of Tween-20 was separately autoclaved and added into the medium and inoculated with 5 mm of mycelia from *A. niger*. After incubation at 25 °C for 5 days, the lipolytic activity was indicated by the appearance of a visible precipitate. All the assays were performed in triplicate.

#### Protease activity

Protease activity of *A. niger* strains was evaluated on YPD agar medium supplemented with 0.4% (w/v) of gelatin (Sigma-Aldrich, USA), and the plates were inoculated with 5 mm of mycelia from strains. After incubation at 25 °C for 5 days, the plates were flooded with saturated aqueous ammonium sulfate. The clear zone around the fungal colony indicated the hydrolysis of gelatin. All tests were realized in triplicate.

### Semiquantification of extracellular enzymes from *A. niger*

#### Inoculum preparation

Five *A. niger* strains (ASP2, ASP6, ASP28, ASP31, and ASP32), selected for their high enzyme production, were initially cultured on PDA medium for 5 days at 25 °C. After incubation, a spore suspension was prepared by flooding the grown fungal cultures with 10 mL sterile distilled water. The spore concentration was adjusted to 5×10^6^ spores/mL using the Thoma cell counting chamber.

### Semiquantification of extracellular enzyme production

An aliquot of 65 μL of the spore suspension of *A. niger* strains was then delivered into the API-ZYM cupules (BioMerieux, France) and incubated at 37 °C for 12 h. One drop of ZYM A (25 g Tris-hydroxymethyl-aminomethane, 11 mL 37% HCl, 10 g sodium lauryl sulfate,100 mL H_2_O) and ZYM B (0.12 g Fast Blue BB, 50 mL methanol, 50 mL dimethylsulfoxide) reagents was added to the cupules, which were placed under white light for 10 min. The API-ZYM test can detect 19 different enzymes and score their concentrations on a rating scale of 0–5. Scoring was done using the API-ZYM® color scale, in which 0 = no enzyme, 1 = 5 nmol, 2 = 10 nmol, 3 = 20 nmol, 4 = 30 nmol, and 5 = 40 nmol or more.

### Quantification of amylase and cellulase activities of *A. niger* strains

#### Measurement of amylase and cellulase activities

The quantitative evaluation of amylase activity was studied in 50-mL flasks containing 25 mL of culture medium. The culture medium composition was (g/L) NaNO_3_ 3 g, MgSO_4_·7 H_2_O 0.5 g, KCl 5 g, KH_2_PO_4_ 1 g, FeSO_4_·7 H_2_O 0.01 g, and CaCl_2_ 0.1 g, supplemented with 1% starch. On the other hand, the cellulase activity was studied on the liquid culture medium described by Hultin and Nordstr6m [10], supplemented with 1% (w/v) carboxymethylcellulose CMC (Sigma Aldrich Co, Germany). These flasks were then autoclaved at 121 °C for 15 min and cooled at room temperature. After sterilization, the flasks were inoculated with a spore suspension of 2×10^5^ spores/mL and incubated for 3 days in an orbital shaker (KS 4000 I control) at 150 rpm. An uninoculated flask was used as a control.

The culture broth was filtered using Whatman filter paper N°1 (Indiamart, India), and then, the filtrate was centrifuged at 8000*g* for 10 min at 10 °C. The culture supernatant (1 mL) was added to 1% (w/v) starch for amylase activity and 1% (w/v) CMC for cellulase activity measurement in 0.05 M sodium acetate buffer (pH 5.6, 8 mL), and incubated at 50 °C for 30 min.

Reducing sugars were determined based on the DNS method [11]. One unit of amylase and cellulase activity (U) is defined as the amount of enzyme that liberated 1 μmol of d-glucose from starch and CMC in a 1 μL reaction mixture under the assay conditions.

### Biomass growth

The fungal biomass was collected, after 7 days of incubation at 25 °C, on Whatman grade 1 filter paper (Indiamart, India), and dried in an oven at 100 °C for 18 h. The biomass of fungal culture was expressed as dry weight (g/L). All the tests were made in triplicate.

### Factors influencing the production of amylase and cellulase enzymes

#### Effect of initial pH on production of amylase and cellulase activities

The effect of initial pH on the production of amylase and cellulase enzymes was evaluated on liquid culture. The medium broth of amylase and cellulase activities was adjusted of the initial pH of 3, 4, 5, 6, 7, and 8 with hydrochloric acid (4 M) and inoculated with 1% (v/v) of *A. niger* ASP2. After inoculation, the cultures were incubated at 25 °C for 7 days. Amylase and cellulase activities were measured. All tests were performed in triplicate.

#### Effect of temperature on production of amylase and cellulase activities

The effect of different temperatures on the production of amylase and cellulase enzymes was evaluated on liquid culture. The medium broth of amylase and cellulase activities was adjusted of the initial pH 7 with hydrochloric acid (4 M) and inoculated with 1% (v/v) of *A. niger* ASP2. After inoculation, the cultures were incubated at different temperatures (25, 28, 30, 35, 40, and 45°C) for 7 days. Amylase and cellulase activities were measured. All tests were performed in triplicate.

#### Effect of incubation period on the production of amylase and cellulase activities

The dynamic of production of amylase and cellulase enzymes by *A. niger* ASP2 strains was evaluated by the measurement of enzymatic activity in different incubation time. *A. niger* ASP2 strains were inoculated with 1% (v/v) in medium broth adjusted in pH 7 with hydrochloric acid (4 M) and cultivated at 25 °C. After 24, 48, 72, 96, 120, 144, and 168 h of incubation, the amylase and cellulase were determined. All assays were performed in triplicate.

### Statistical analysis

Means were based on three replications. The values of different parameters were expressed as the mean ± standard deviation. Student-Newman-Keuls test was performed using the statistical analysis package SPSS 10 for Windows (SPSS Inc., Chicago, USA) at *p*<0.05, to evaluate the significance of differences between mean values.

## Results

### Qualitative of extracellular enzymes from *A. niger*

**Fig. 1:**
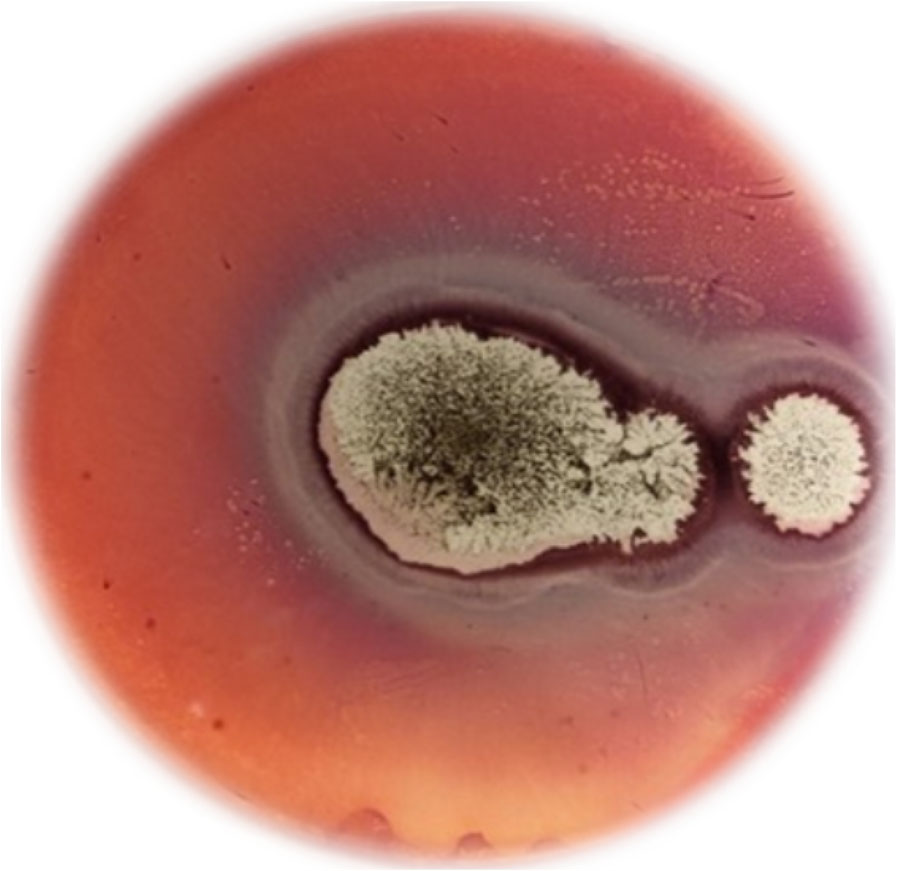
Cellulase activity of *Aspergillus niger* in plate agar medium

The production of several enzymes by *A. niger* was detected in an agar medium containing a different carbon source, and the result is reported in Table 1 and Fig. 1. *A. niger* strains demonstrated the amylase, cellulase, and lipase activities by the hydrolysis of starch, cellulose, and Tween-20, respectively. Also, the amylase and cellulase activities dominated compared with the lipase activity in the percentage of strains. However, no protease activity was detected from all *A. niger* strains. Furthermore, the amylase, cellulase, and lipase activities were detected at different levels in strains from all date by-products. Hence, the *A. niger* strains isolated from date fruit, date paste, and date-seed-powder showed high productions of amylase and cellulase and to some degree lipase, to those isolated from date juice. On the other hand, the absence of enzyme production (i.e., protease) may be undetectable, or the reaction is not an absolute confirmation of a species inability to produce this particular enzyme, which could justify the non-detection of a protease enzyme on all strains tested despite the positive growth on this media. It could also be due to the ability of the fungus to use other materials in the medium other than the tested substrates.

**Table 1:** Hydrolytic activity (mm) of *Aspergillus niger* isolates, obtained from different date by-products

*A.niger* isolates	Source	Diameters (mm)			
		Starch	Gelatin	Cellulose	Tween-20
ASP1	Date paste	8.3±0.58^c^	-	4.3±0.58^a^	4.3±0.58^b^
ASP2		10.6±0.58^d^	-	10.3±0.58^b^	5.3±0.58^b^
ASP3		4.3±0.58^b^	-	4.6±0.58^a^	-
ASP4		-	-	4.3±0.58^a^	4±1.00^b^
ASP5	Date fruit	8.3±0.58^e^	-	5.3±0.58^c^	-
ASP6		9±0.00^e^	-	5.6±0.58^d^	-
ASP7		4.3±0.58^b^	-	5.6±1.15^d^	-
ASP8		3.3±0.58^b^	-	3±1.00^b^	-
ASP9		-	-	-	5.3±0.58^e^
ASP10		-	-	5.3±0.58b^c^	-
ASP11		6.3±1.15^d^	-	6.3±0.58^d^	3.3±0.58^b^
ASP12		3.3±1.15^b^	-	-	-
ASP13		-	-	-	-
ASP14		-	-	4.3±0.58^bc^	3.3±0.58^b^
ASP15		-	-	-	-
ASP16		-	-	6.3±0.58^d^	-
ASP17		-	-	6.3±0.58^d^	3±0.58^bc^
ASP18		5.3±0.58^c^	-	-	-
ASP19		-	-	-	-
ASP20		3.6±0.58^b^	-	5.3±0.58^bc^	4±0.00^d^
ASP21		8±1.00^e^	-	-	-
ASP22		-	-	-	2.6±0.58^b^
ASP23		8.3±0.58^e^	-	-	-
ASP24		-	-	-	3.3±0.58^b^
ASP25		-	-	-	-
ASP26	Date-seed-powder	7.3±0.58^c^	-	6±0.00^b^	3.3±0.58^b^
ASP27		2.6±0.58^a^	-	7.3±0.58^bc^	5.3±0.58^c^
ASP28		3±0.00^a^	-	8.3±0.58^cd^	-
ASP29		7.6±1.15^c^	-	4±0.00^a^	-
ASP30		5.3±0.58^b^	-	6.3±0.58^b^	-
ASP31		9.3±0.58^d^	-	6±1.00^b^	5±0.00^c^
ASP32		7.3±0.58^c^	-	9.3±0.58^d^	3.3±0.58^b^
ASP33	Date-flesh-powder	4.6±0.58^c^	-	-	-
ASP34		4±0.00^b^	-	-	-
ASP35		-	-	3.6±0.58^b^	3.6±0.58^b^
ASP36		-	-	-	-
ASP37	Date juice	-	-	-	-
ASP38		3±1.00^b^	-	-	-
ASP39		-	-	-	4.3±0.58^b^
ASP40		-	-	-	-

Results are means ± SD (*n*= 3). Values of the same column, followed by the same letter, are not statistically different (*p* < 0.05) as measured by Student-Newman-Keuls test

“-” indicates no detected activity

Out of forty isolates, the strains ASP2, ASP6, and ASP31, and ASP2, ASP28, and ASP32, showing high activity for amylase (10.6, 9.3, and 9 mm) and cellulase (10.3, 9.3, and 8.3 mm) (Table 1), respectively, were selected for the semiquantitative analysis for enzyme production. The *Aspergillus* strains isolated from date by-products may be considered as a good source of enzymes (amylase, cellulase, and lipase).

### Semiquantification of extracellular enzymes from *A. niger*

The extracellular enzymatic activity of *Aspergillus niger* strains obtained by the API-ZYM system is reported in Table 2. The reactions obtained showed that all 5 strains shared almost similar enzymatic activities. Hence, lipase (C14), cystine arylamidase, trypsin, β-glucuronidase, α-mannosidase, and α-fucosidase were uniformly absent in all the isolates, while all isolates showed strong activity of phosphatase acid and Naphthol-AS-BI-phosphohydrolase, intermediate levels of phosphatase alkaline, esterase (C4), esterase lipase (C8), and leucine arylamidase. These fungi were characterized by the production of some enzymes taking part in carbohydrate hydrolysis (α-glucosidase, β-glucosidase, N-acetyl-β-glucosaminidase), and to a lower degree α-galactosidase and β-galactosidase enzymes. *Aspergillus niger* ASP2 was selected for their high production of various enzymes and used in a future experiment.

**Table 2:** Enzymes released by *Aspergillus niger* detected by the APY-ZYM® system

Enzymes	Strains				
	ASP2	ASP6	ASP28	ASP31	ASP32
Phosphatase alkaline	5	1	5	4	1
Esterase (C4)	5	2	5	4	2
Esterase lipase (C8)	5	2	5	4	1
Lipase (C14)	0	0	0	0	0
Leucine arylamidase	4	2	3	2	1
Valine arylamidase	2	0	0	0	0
Cystine arylamidase	0	0	0	0	0
Trypsin	0	0	0	0	0
Chymotrypsin	2	0	0	2	0
Phosphatase acid	5	5	5	4	4
Naphthol-AS-BI-phosphohydrolase	5	4	4	5	3
α-Galactosidase	2	1	0	1	0
β-Galactosidase	2	1	0	0	0
β-Glucuronidase	0	0	0	0	0
α-Glucosidase	3	1	2	2	0
β-Glucosidase	5	4	4	4	3
N-acetyl-β-glucosaminidase	4	5	4	4	3
α-Mannosidase	0	0	0	0	0
α-Fucosidase	0	0	0	0	0

The scale of the API-ZYM® test was used for enzyme quantification, with 0=not detected activity, 1=5 nmol substrate metabolized, 2=10 nmol, 3=20 nmol, 4=30nmol, and 5 ≥40 nmol

### Quantitative evaluation of amylase and cellulase activities

#### Quantification of amylase and cellulase activities

The quantification of amylase and cellulase activities from *A. niger* ASP2 was measured in the supernatant by the DNS method. The result demonstrated the ability of *A. niger* ASP2 to produce amylase and cellulase enzymes with activities of 8.37±0.09 and 1.76±0.09 U/mL, respectively. On the other hand, the biomass growth after 7 days of incubation at 25 °C in amylase and cellulase activities was 1.17±0.10 and 1.47±0.09 g/L, respectively. The amylase activity of *A. niger* ASP2 was significantly (*p*<0.05) higher than of the cellulase activity.

#### Effect of initial pH

**Fig. 2:**
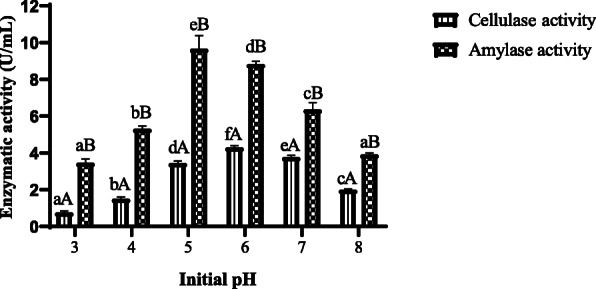
Effect of initial pH on production of amylase and cellulase enzymes by *A. niger* ASP2. Values are mean ± standard error of triplicates. a–f Means in same column of each parameter with different lower case letters differed significantly (*p* < 0.05). A, B Values in the enzymatic activity (cellulase and amylase) for each parameter with different lower case letters differed significantly (*p* < 0.05)

The results of the levels of amylase and cellulase activities obtained in different initial pH are presented in Fig. 2. The maximum level of amylase (9.63 U/mL) was reached at pH 5, while 4.21 U/mL was obtained for cellulase at pH 6 from *A. niger* ASP2. The amylase activity of *A. niger* ASP2 was significantly (*p*<0.05) higher than of the cellulase activity in the all initial pH studied.

#### Effect of temperature

**Fig. 3:**
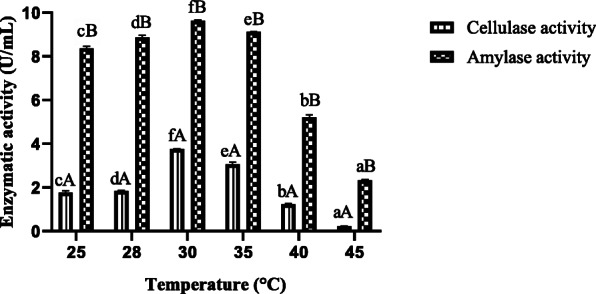
Effect of incubation temperature on production of amylase and cellulase enzymes *A. niger* ASP2. Values are mean ± standard error of triplicates. a–f Means in same column of each parameter with different lower case letters differed significantly (*p* < 0.05). A, B Values in the enzymatic activity (cellulase and amylase) for each parameter with different lower case letters differed significantly (*p* < 0.05)

The amylase and cellulase activities obtained at different temperatures of incubation from *A. niger* ASP2 are reported in Fig. 3. The maximum production of cellulase and amylase enzymes for ASP2 strains tested were recorded at 30 °C with 3.76 U/mL of cellulase and 9.5 U/mL of α-amylase productions (Fig. 3). The amylase and cellulase activities of *A. niger* ASP2 at 30 °C were significantly (*p*<0.05) higher than other temperatures studied. Moreover, the amylase activity was significantly (*p*<0.05) higher than of the cellulase activity in all temperatures values. This activity decreases drastically when the incubation temperature exceeded 40 °C. This shows clearly that the production of these enzymes by *A. niger* strains is greatly affected by this parameter.

#### Effect of incubation time

**Fig. 4:**
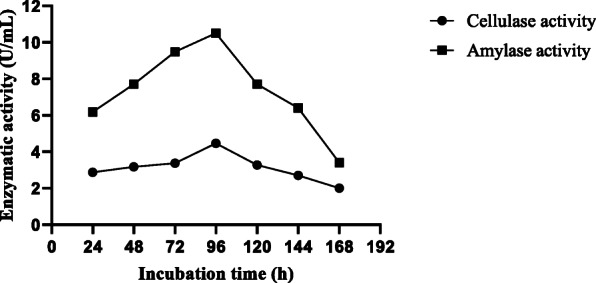
Effect of incubation time on production of amylase and cellulase enzymes *A. niger* ASP2

The effect of incubation time in the production of amylase and cellulase activities of *A. niger* ASP2 reached their maximum after 96 h of fermentation at 25 °C (Fig. 4). The activities of amylase and cellulase production were 10.50 and 4.47 U/mL, respectively.

## Discussion

The *Aspergillus* strains isolated from date by-products demonstrated the ability to produce various enzymes such as amylase, cellulase, and lipase. The studies of Mostafa et al. [8] and Sattar et al. [12] demonstrated the ability of *Aspergillus* to produce several enzymes. The synthesis of enzymes may also be due to the mycelial condition/age [13], and the type of enzyme produced may vary with the environmental conditions, particularly the type of substrate [14].

The semiquantitative enzyme production demonstrated the important enzymatic profile of selected *Aspergillus* strains from date by-products. Variable results measured by API-ZYM tests have been reported in other filamentous fungi, including *Penicillium* and *Verticillium* isolates [15, 16]. This result indicates that the enzymes produced by *A. niger* are extracellular.

The initial pHs 5 and 6 showed the high production rate of amylase and cellulase enzymes produced by *A. niger* ASP2. Similar results are obtained with *A*. *ochraceus* and *A*. *niger* [17, 18]. Other authors reported optimal acidic pHs for amylases from *A. niger* [19, 20]. The initial pH of 5.5 showed the high production of cellulase enzyme from fungi [8]. The pH plays an important role in microbial growth, and the change in pH from the optimum to extreme levels results in the inactivation of the enzymes of the organisms which hinders saccharification [21]. This finding is important to the fermentation process because of its contribution to the inhibition of contaminations caused by neutrophil bacteria.

The temperature of the high production of amylase and cellulase enzymes was demonstrated at 30 °C. These results are in agreement with Varalakshmi et al. [22] indicating that the best enzyme production in *A. niger* is obtained at room temperature both in submerged fermentation (SmF) and solid-state fermentation (SSF). Various studies reported that this parameter is very important during the enzymatic production, and they found that 30 °C is the optimal temperature for some fungus, especially *Aspergillus* species [23, 24]. However, the optimal production of crude cellulase was determined at 40 °C [25]. Also, Mostafa et al. [8] demonstrated that the production of cellulase from fungus was obtained at 60 °C. On the other hand, the amylase production from *A. clavatus* achieved the maximum at 30 °C [9]. The temperature plays an important factor in the production of amylase and cellulase enzymes.

The maximum production level of amylase and cellulase enzymes was detected after 96 h of fermentation at 25 °C. This result is in agreement with previous studies reported by Acharya et al. [26] and Devanathan et al. [27]. This finding was comparable to those obtained from *A. clavatus* [9]. However, Sulyman et al. [25] reported that the maximum production of cellulase was detected at 120 h. The decreased activity obtained after this period was probably due to catabolite repression by glucose released from starch and CMC hydrolysis. It can also be due to the depletion of nutrients, lag phase of fungi, and production of proteases in the fermentation medium [28].

*A. niger* ASP2, isolated from date paste, demonstrated a high and important production of several enzymes, including amylase and cellulase enzymes. The study of some parameters (pH, T °C, and incubation time), influencing the maximum production of amylase and cellulase enzymes, this finding could be used in the valorization and the production of enzymes by fermentation of different agro-industrial residues.

## Conclusion

*Aspergillus niger* ASP2 strain isolated from date by-product demonstrated the production of extracellular enzymes such as amylase, cellulase, and lipase. *A. niger* ASP2 strain isolated from date paste showed the high production rate of these enzymes (amylase, cellulase, and lipase) in plate agar medium. Therefore, the highest production was recorded in initial pH 5 and pH 6 for amylase and cellulase, respectively. The maximum production of amylase and cellulase enzymes was demonstrated at a temperature of 30 °C and after 96 h of incubation. This fungus isolated from undervalued date by-products can produce several extracellular enzymes using the carbon source contained in different agroindustrial residues.

## Data Availability

All data generated or analyzed during this study are included in this article.

## Abbreviations

NaCl: Sodium chloride
pH: Hydrogen potential
UV: Ultraviolet
TDF: Total dietary fibers
PDA: Potato dextrose agar
CMC: Carboxymethylcellulose
